# The Impact of Antimicrobial Stewardship Program on Injudicious Use of Cefuroxime

**DOI:** 10.3389/fphar.2020.565818

**Published:** 2021-02-08

**Authors:** Ann L. Arulappen, Monica Danial, Norliza Haron, Lim Choo Hau, Amer Hayat Khan

**Affiliations:** ^1^ Discipline of Clinical Pharmacy, School of Pharmaceutical Sciences, Universiti Sains Malaysia, Georgetown, Malaysia; ^2^ Pharmacy Department, Hospital Seberang Jaya, Georgetown, Malaysia; ^3^ Clinical Research Center, Hospital Seberang Jaya, Georgetown, Malaysia; ^4^ Infection Control Unit, Hospital Seberang Jaya, Georgetown, Malaysia

**Keywords:** cefuroxime, defined daily dose, antimicrobial stewardship, perioperative antimicrobial prophylaxis, injudicious

## Abstract

Antimicrobial stewardship (AMS) program promotes the judicious use of antimicrobials. Hence, this study was conducted to analyze the impact of stewardship on the prescribing pattern of cefuroxime injection among the surgeons as perioperative antimicrobial prophylaxis (PAP). This study was conducted retrospectively in Malaysia. Various outcomes were measured including cefuroxime usage, compliance with the guidelines, surgical site infections, and cost savings. A total of 1,601 patients were recruited in the study. In terms of usage, the total defined daily dose (DDD) prior to the intervention was 202 DDD/100 procedures compared to that after intervention which was 144 DDD/100 procedures (*p* < 0.05). On the other hand, the excessively long administration of PAP dropped from 94.4 to 30.3% (*p* < 0.001). Focusing on the compliance with the newly developed local guidelines, it has increased from 53 to 94.3% after the interventions were made (*p* < 0.001), whereas the rate of surgical site infections was reduced from 17.0 to 9.0%. The cost of antibiotic being used has significantly reduced after the study intervention (*p* = 0.007). The quality of PAP directly impacts the antimicrobial usage, the surgical site infections, and the total cost involved. Thus, it is crucial to maintain the standard of PAP at all times in healthcare settings.

## Introduction

Globally, the irrational use of antibiotics constitutes an ultimate factor related to the development of antibiotic resistance which in turn necessitates the enforcement of antimicrobial stewardship programs in the healthcare. The judicious use of antibiotics has a significant impact on the healthcare. Monitoring antibiotic usage is a recommended component of antimicrobial stewardship programs, providing information about the pattern and trends of usage at intra-facility and inter-facility level. Consequently, these data would provide evidence of injudicious use and reflects the necessity to conduct additional audit or interventions.

Defined daily dose (DDD) had been in use to standardize the unit of comparison worldwide. It is the average of the maintenance dose of a single antibiotic in its main indication for adults per day. According to the World Health Organization (WHO), each drug has an anatomical therapeutics chemical (ATC) code and a DDD value in grams (World Health Organization, 2016). It was recommended to express DDD per 100 bed-days in hospitals and DDD per 1,000 inhabitant-days for outpatients as to express the actual usage (Hutchinson et al., 2004; Dellit et al., 2007; Pope et al., 2009).

Cefuroxime, grouped as second-generation cephalosporins, has always been the preferred choice of antibiotic especially by the orthopedic surgeons. It has been used for various indications, including surgical prophylaxis, compound fractures, or even simple abrasion wound. Cefuroxime usage had increased drastically in these past three years at this study center which is a major specialist hospital. The DDD has overtaken the major tertiary centers in Malaysia based on the national data.

In a recent point prevalence survey (PPS) conducted, it was estimated that 85% of antibiotics use was inappropriate when compared to the local guidelines. Prolonged administration of perioperative antimicrobial prophylaxis (PAP) for a longer time than necessary is associated with increased risk of surgical site infection (SSI) and further burdens the healthcare costs (Burke, 2001; van Kasteren et al., 2003).

Consequently, there is a threatening rise in bacterial resistance trend with the apparent drop in the numbers of antimicrobials newly sanctioned into the market (Boucher et al., 2009; Society for Healthcare Epidemiology of America et al., 2012). Hence, the antimicrobial stewardship (AMS) program has been enforced to warrant the judicious use of antimicrobials, decrease overutilization of antimicrobial agents, and combat the development of resistance (MacDougall and Polk, 2005; Owens, 2008).

Injudicious use of antibiotics may lead to increased risk of SSI. SSI was classified based on the USA National Research Council’s modified wound classification criteria (Mangram et al., 1999). Generally, prophylactic antibiotic will not be given for clean surgical procedures, whereas it is only recommended in procedures involving prostheses so as to prevent serious complications that may occur thereafter.

This study illustrates the impact of strict stewardship implemented and changes made on the policy pertaining to the supply of cefuroxime by the Pharmacy Department in a major specialist hospital.

## Methods

### Study Location

This single center study was carried out at a major specialist hospital in Penang, Malaysia, which serves a population of approximately 900,000 patients. This hospital has 393-bed occupancy capacity and two intensive care units accommodating both adults and pediatrics. This facility provides acute, general medicine, surgery, and maternity plus intensive care services. Ethical board approval by MREC was obtained prior to the initiation of this study.

### Data Collection

Cefuroxime data were collected retrospectively. Pre-intervention data includes the period from May 2019 to October 2019, whereas post-intervention data includes the period from November 2019 to April 2020. Only prescriptions from orthopedic wards involving patients >15 years old scheduled for any type of surgery and started with cefuroxime injection as PAP were included in this study. Those patients with underlying preexisting infectious conditions, such as osteomyelitis, were excluded from this study. The data were conveyed to an Excel worksheet using coding system. The data includes patient demographics, admission and discharge dates, date of surgery, type and duration of surgery, wound contamination classification, total number of prophylactic doses, duration of antibiotic prophylaxis, and any infection at the surgical site. The required information is obtained from the operation notes and patients’ record files.

The World Health Organization 2013 Guidelines for Anatomical Therapeutic Chemical/Defined Daily Dose (ATC/DDD) were used in order to compute the DDD. The DDD was calculated as the total number of grams administered per year which was further divided by the WHO DDD in grams (Hutchinson et al., 2004). Thus, the DDD is an approximation of the number of days of antibiotic therapy.

### Tools Development

A new local guideline was developed with consent from the Head of the Orthopedic Department for therapeutic use and surgical prophylaxis. A number of policy changes were made involving antibiotic prescribing and indenting policies. The new prescribing policy allowed only the medical officers and specialists to prescribe cefuroxime injection, whereas the indenting policy reflects the necessity to fill in the Injection Antibiotic Order Form together with the prescription to be sent to the Pharmacy Department to get the supply of antibiotic. The purpose of filling in the Injection Cefuroxime Antibiotic Order Form was to enable us to further analyze the indication, dose, and duration of the antibiotic prescribed concomitantly to reflect the compliance with the new local guideline published.

### Outcome Measurement

The usage of cefuroxime injection was determined through the quantitative calculation in terms of DDD and grams (g). This method of consumption calculation allows a direct comparison with the other facilities.

The compliance with PAP in terms of appropriateness and duration was determined by trained pharmacists in accordance with the newly developed local guideline. If PAP was in compliance completely with the locally developed guideline, it was appraised as appropriate, whereas if it was not fulfilling the criteria, then it was categorized as inappropriate.

SSI was classified based on the USA National Research Council’s modified wound classification criteria (Mangram et al., 1999). No prophylactic antibiotic treatment is indicated for all the clean surgical procedures. The diagnosis of SSI was made in accordance with the criteria of the US Centers for Disease Control and Prevention (CDC) (Mangram et al., 1999). The rate of SSI was assessed according to the clinic records within 6 months after operation.

The cost estimated in this study was solely based on the antibiotic usage only. It does not include the other hospital charges such as room price, surgery fees, and facilities used. The cost of the antibiotic used before and after the intervention period was calculated in Ringgit Malaysia (RM).

### Statistical Analysis

Descriptive statistical analysis was performed using SPSS. The categorical data during the pre- and post-intervention periods were related using the Chi-square test. *t*-Test was deployed to calculate the changes in consumption. The 95% confidence interval (CI) was calculated for antimicrobial consumption. A *p*-value of <0.05 was considered significant.

## Results

Throughout the 6-month pre-intervention period, a total of 1,189 patients underwent orthopedic related surgeries, and approximately 846 of them were recruited in this study with 421 (49.8%) males and 425 (50.2%) females. Meanwhile, 755 out of 1,201 patients were recruited during the post-intervention period with 399 (52.8%) males and 356 (47.2%) females. All the patients recruited in this study were found to fulfill the study criteria for inclusion. The demographic characteristics of the patients are tabulated in Table 1. Further analysis is depicted in Table 2.

**TABLE 1:** Demographic characteristics of study patients.

Variables	Pre-intervention, (n (%))	Post-intervention, (n (%))
Males	421 (49.8%)	399 (52.8%)
Females	425 (50.2%)	356 (47.2%)
Age (years)	45 (±18.9)	43 (±18.6)

**TABLE 2:** Univariate analysis between pre-intervention and post-intervention of various variables used to evaluate the impact of antimicrobial stewardship program.

Variables	Pre-intervention, (n (%))	Post-intervention, (n (%))	Comparison^a^	*p-value*
Gender	—	—	—	0.229
Male	421 (49.8)	399 (52.8)	—	—
Female	425 (50.2)	356 (47.2)	—	—
Age (years)^b^	45 (±18.9)	43 (±18.6)	—	0.014
Defined daily dose^c^	202 (191.0,207.0)	144 (191.0,205.0)	—	0.004
Usage (gram)^c^	1,524 (1,440.0,1561.5)	1,154 (1,479.0, 1,558.5)	—	0.007
Appropriateness for PAP	846	755	—	0.046
Yes	448 (53.0)	712 (94.3)	—	—
No	398 (47.0)	43 (5.7)	—	—
Duration of PAP	—	—	—	<0.001
i. Single dose	21 (2.5)	59 (7.8)	i vs. ii	0.988
ii. < 24 h	26 (3.1)	467 (61.9)	ii vs. iii	<0.001
iii. > 24 h	799 (94.4)	229 (30.3)	i vs. iii	<0.001
Cost^c^	RM 13548.36 (RM 12801.60, RM 13881.74)	RM 10254.62 (RM 5367.40, RM 10841.36)	—	0.007
Surgical site infections	—	—	—	—
Clean	702	691	—	<0.001
Contaminated	144	64	—	—
Reduction trend (slope)^b^	−0.72 (±9.33)	−10.14 (±82.30)	—	0.821

Abbreviation: PAP, perioperative antimicrobial prophylaxis.

a Comparison based on Tukey HSD post hoc test.

b Reported as mean (±SD).

c Reported as median (Q1,Q3).

### Usage of Antibiotics

The total DDD prior to the intervention was 202 DDD/100 procedures compared to that after intervention which was 144 DDD/100 procedures (*p* < 0.05). The ratio was 2 DDD and 1.5 DDD for a single procedure, respectively. In terms of grams, a total of 1524 g was used prior to the intervention and 1154 g was used after intervention. Approximately, 25% of total usage reduction was noted. In terms of reduction trend (slope), acceptable antibiotic usage reduction is obtained after intervention compared to before intervention.

### Evaluation of PAP

Before the intervention, 846 (100%) patients were given PAP and 89 (10.5%) patients had no indication for it. After the intervention, 755 (100%) patients received PAP and 35 (4.6%) had no indication for it. The route of administration was intravenous for all the procedures performed during both pre- and post-intervention. PAP was administered within an hour before the incision. The appropriateness was 448 (53%) prior to intervention and 712 (94.3%) after intervention.

### Duration of PAP

In the majority of cases, PAP was used for a long period of time inappropriately. Precisely, there were 799 (94.4%) out of 846 patients deemed to be using PAP inappropriately in the pre-intervention phase and 229 (30.3%) out of 755 patients in the post-intervention phase. This difference was statistically significant (*p* < 0.001).

### Surgical Site Infections

Prior to the intervention phase, 702 cases were reported as clean, whereas the remaining 144 cases were reported as contaminated. However, post-intervention total clean cases reported were 691 and the number of contaminated cases reduced to 64. This finding was statistically significant (*p* < 0.001).

### Cost Savings

The cost of antibiotic being used was significantly reduced after the study intervention. The total cost of antibiotic was RM 13,548.36 prior to intervention and RM 10,254.62 after intervention. Approximately, 14% of cost savings were calculated after the intervention was made (*p* = 0.007).

### Reduction Trend (Slope)

The slope was −0.72 during the pre-intervention period and −10.14 during the post-intervention period. This has revealed that the usage of cefuroxime injection has declined remarkably after the intervention was made (*p* = 0.821).

## Discussion

Our data revealed that cefuroxime consumption had reduced significantly after the implementation of antimicrobial stewardship strategies to raise the quality of perioperative antimicrobial prophylaxis (PAP). A collaborative approach between the orthopedic surgeons and antimicrobial stewardship team was made to overcome this issue. Various interventions were implemented, including local guideline development, Injection Cefuroxime Antibiotic Order Form, and antibiotic formulary restriction.

It is crucial to calculate the antibiotic consumption for PAP using the ATC/DDD system in order to attain a general standardization as to equate antibiotic usages in other healthcare centers. The antibiotic usage at our hospital prior to the intervention was 202 DDD/100 procedures, which is higher compared to the usage in other hospitals locally (157 DDD/100 procedures) based on the national data. In the post-intervention phase, a statistically significant reduction of usage up to 144 DDD/100 procedures was noted (*p* < 0.05). Similarly, two published studies reported significant reduction in antibiotic usage. One study was conducted in Japan and stated that antibiotic usage reduced from 160.6 to 129 DDD/100 procedures (Takahashi et al., 2010). The other study was conducted in Germany and specified that antibiotic usage had reduced from 121 to 79 DDD/100 procedures (van Kasteren et al., 2005).

Many studies presented the use of first or second generation of cephalosporins as surgical prophylaxis (Mangram et al., 1999; Bratzler and Houck, 2005). Looking at the appropriateness of PAP perspective, the proportion of appropriate antibiotic choice significantly amplified from 53 to 94.3% after the interventions were made. The appropriateness in this study was measured in terms of adherence to our newly established local antibiotic guideline, as part of the antimicrobial stewardship strategy. The local guideline was developed based on the Malaysian National Antibiotic Guideline, and certainly some modifications were made to fit into our local antibiogram (Ministry of Health Malaysia, 2019). This finding is similar to the study conducted in Turkey whereby the proportion of appropriate antibiotic choice amplified from 77.6 to 90.6% after the intervention (*p* < 0.001) (Bozkurt et al., 2013).

Additionally, some studies have reported that the majority of the surgeons would prefer to extend the duration of antimicrobial prophylaxis longer than the recommended period (Hutchinson et al., 2004; Muller et al., 2006; World Health Organization, 2016). In this study, the degree of compliance in terms of duration of PAP was from 5.6% before intervention to 69.7% after intervention, which was statistically significantly (*p* < 0.001). However, the same compliance rate was stated to be 65.8–82% in quite a number of countries (Muller et al., 2006; Owens, 2008). This clearly reveals that the extended length of the treatment remains as the major unresolved problem worldwide.

Prevention of SSI was considered as the core challenge for the surgeons. A number of journals have revealed that up to 70% of SSI may be avoided through the administration of antimicrobial prophylaxis at the correct moment and at the correct dose (van Kasteren et al., 2003). Maintaining aseptic conditions and giving the right choice of antibiotic as surgical prophylaxis are the crucial strategies in preventing infections at surgical site. It is proven that strict adherence to the newly implemented local guideline on the choice of antibiotic has reduced the rate of SSI from 17 to 9% (*p* < 0.001). This finding is almost similar to another published study whereby the rate of SSI reduced from 18.5 to 12% (*p* < 0.001) (Bozkurt et al., 2013).

Improvement in the quality of the antibiotic prophylaxis has also a significant impact on the cost involved in the overall healthcare system. Approximate reduction of 14% in cost was obtained in this study based on the antibiotic usage. Financial burden was certainly reduced significantly in this facility. A further detailed study should be carried out to analyze the direct and indirect cost savings resulting from the interventions made. Various studies have proven that improving the quality of antibiotic prophylaxis has greater impact on total cost savings (van Kasteren et al., 2005; Bozkurt et al., 2013).

## Conclusion

The study has proven that, with the implementation of a local guideline and compliance, frequent audits on the perioperative antibiotic prophylaxis and a good collaboration among the healthcare professionals have successfully improved the judicious use of cefuroxime as surgical prophylaxis.

### Strengths and Limitations

This study center conducts all the major and complicated orthopedic surgeries, and the surgeons are well-experienced professionals. This is the major strength of this study. On top of that, another added advantage is the number of surgeries conducted which is as equal as other tertiary care centers in Malaysia.

However, this study has its own drawbacks as well. The most significant drawback is that this study was conducted at a single center only. Secondly, there is no permanent infectious disease physician in this center. We have only visiting ID physicians twice in a month. Thus, establishing a local guideline and communicating with the orthopedic surgeons were quite challenging.

## Data Availability

The raw data supporting the conclusions of this article will be made available by the authors, without undue reservation.
